# Associations of metal mixtures with metabolic-associated fatty liver disease and non-alcoholic fatty liver disease: NHANES 2003–2018

**DOI:** 10.3389/fpubh.2023.1133194

**Published:** 2023-03-06

**Authors:** Zhilan Xie, Ruxianguli Aimuzi, Mingyu Si, Yimin Qu, Yu Jiang

**Affiliations:** School of Population Medicine and Public Health, Chinese Academy of Medical Sciences and Peking Union Medical College, Beijing, China

**Keywords:** metabolic associated fatty liver disease, non-alcoholic fatty liver disease, Bayesian kernel machine regression, quantile-based g-computation, heavy metals

## Abstract

**Objective:**

The hepatotoxicity of exposure to a single heavy metal has been examined in previous studies. However, there is limited evidence on the association between heavy metals mixture and non-alcoholic fatty liver disease (NAFLD) and metabolic-associated fatty liver disease (MAFLD). This study aims to investigate the associations of 13 urinary metals, individually and jointly, with NAFLD, MAFLD, and MAFLD components.

**Methods:**

This study included 5,548 adults from the National Health and Nutrition Examination Survey (NHANES) 2003–2018. Binary logistic regression was used to explore the associations between individual metal exposures and MAFLD, NAFLD, and MAFLD components. Bayesian kernel machine regression (BKMR) and Quantile-based g-computation (QGC) were used to investigate the association of metal mixture exposure with these outcomes.

**Results:**

In single metal analysis, increased levels of arsenic [OR 1.09 (95%CI 1.03–1.16)], dimethylarsinic acid [1.17 (95%CI 1.07–1.27)], barium [1.22 (95%CI 1.14–1.30)], cobalt [1.22 (95%CI 1.11–1.34)], cesium [1.35 (95%CI 1.18–1.54)], molybdenum [1.45 (95%CI 1.30–1.62)], antimony [1.18 (95%CI 1.08–1.29)], thallium [1.49 (95%CI 1.33–1.67)], and tungsten [1.23 (95%CI 1.15–1.32)] were significantly associated with MAFLD risk after adjusting for potential covariates. The results for NAFLD were similar to those for MAFLD, except for arsenic, which was insignificantly associated with NAFLD. In mixture analysis, the overall metal mixture was positively associated with MAFLD, NAFLD, and MAFLD components, including obesity/overweight, diabetes, and metabolic dysfunction. In both BKMR and QGC models, thallium, molybdenum, tungsten, and barium mainly contributed to the positive association with MAFLD.

**Conclusion:**

Our study indicated that exposure to heavy metals, individually or cumulatively, was positively associated with NAFLD, MAFLD, and MAFLD components, including obesity/overweight, diabetes, and metabolic dysfunction. Additional research is needed to validate these findings in longitudinal settings.

## 1. Introduction

Non-alcoholic fatty liver disease (NAFLD), the leading cause of cirrhosis and hepatocellular carcinoma, prevails worldwide with an estimated prevalence of 32.4% (1). The prevalence of NAFLD has increased parallel to the increasing prevalence of obesity, type 2 diabetes, and other metabolic syndromes (2). Recently, experts from European Liver Patients Association (ELPA) proposed a new nomenclature, metabolic associated fatty liver disease (MAFLD), to replace the earlier NAFLD term (3). The new nomenclature changes the emphasis from excluding other liver diseases or excessive alcohol consumption to identifying cases with concomitant metabolic dysfunction (4). This change in definition affects the prevalence, risk factors, and outcomes of these two diseases (5, 6). The pooled prevalence of MAFLD was reported to be 39.22%, which was higher than that of NAFLD at 33.86% (5). Compared with NAFLD, MAFLD tended to be more closely associated with obesity, diabetes, and high fibrosis scores (5). Emerging data have also suggested that patients with MAFLD tended to have higher risks of cardiovascular disease and all-cause mortality than those with NAFLD (6). Furthermore, similar to the increasing trend of NAFLD, the prevalence of MAFLD in the United States also increased from 34.4% in 2011 to 38.1% in 2018 (7). Given their increasing prevalence and adverse outcomes, identifying determinants of MAFLD and NAFLD is of substantial public health interest.

NAFLD and MAFLD are heterogeneous disorders with genetic and environmental factors involved in their pathogenesis and progression. Beyond dietary factors and physical activity, previous animal and human studies suggested that heavy metals may play essential roles in the etiology of NAFLD and MAFLD (8, 9). Heavy metals are metallic elements with high density and atomic weight and have adverse health impacts on humans (10). Heavy metal exposure is widespread in humans due to its various sources, including the atmosphere, domestic effluents, industrial waste, and agriculture (11, 12). Heavy metals threaten human health because they are non-biodegradable and can be deposited in body tissues or organs to produce harm after initial exposure (13). Toxicology studies have shown that heavy metals (lead, cadmium, and arsenic) could disturb the hypothalamic dopaminergic system and endoplasmic reticulum proteostasis (14), impair adipogenesis and adipocytokines secretion, and induce hepatic inflammation and steatosis (15, 16). In addition, exposure to heavy metals was a risk factor for many metabolic abnormalities, such as diabetes (17), metabolic syndrome (18), obesity, and hypertension (17). Several previous studies reported positive associations between mercury (19), arsenic (20), lead (9), cadmium (21), and metal mixture (22, 23) with NAFLD. However, the evidence of the association between heavy metals and the risk of MAFLD is limited. The exact physiological roles of other metals in MAFLD and NAFLD patients are still unknown. Additionally, previous studies on metals generally evaluated the influence of single metals, but this approach could not reflect the reality that individuals are exposed to multiple metals simultaneously.

To fill these knowledge gaps, we conducted this study among US adults who participated in the National Health and Nutrition Examination Survey (NHANES) 2003–2018 survey cycles to examine the associations of urinary metal mixtures and individual metals with MAFLD, NAFLD, and MAFLD components using Bayesian Kernel Machine Regression (BKMR) and Quantile based g-computation (QGC). Our study might be informative and instructive for MAFLD and NAFLD etiology and prevention.

## 2. Materials and methods

### 2.1. Study population

The NHANES examines a representative sample of the resident population across the United States, combining interviews and physical examinations (24). Written informed consent was obtained from each participant, and the NHANES protocol was approved by the National Center for Health Statistics (NCHS) Institutional Review Board.

The current study is based on an analysis of data from the combined eight continuous NHANES survey cycles (2003–2018). Two earlier cycles of NHANES (1999–2000 and 2001–2002) were not included because arsenic species were not measured in those cycles. Urinary measurements of heavy metals were taken from 14,058 adults 20 years of age or older. We excluded 8,510 participants due to the missing data in (1) the calculation of the U.S. fatty liver index (USFLI) (remaining, *n* = 6,068), (2) covariates (i.e., ratio of family income to poverty, education, smoking status, and physical activity) (remaining, *n* = 5,548), leaving 5,548 participants for the analyses of MAFLD. The NAFLD analysis sample additionally excluded individuals with excessive alcohol consumption (*n* = 725), positive HBV surface antigen (*n* = 29), and positive HCV RNA (*n* = 66). The final sample size for NAFLD analyses was 4,750. The flow chart of the inclusion and exclusion criteria for the sample population was presented in Supplementary Figure 1.

### 2.2. Measurements of heavy metals

A total of 14 heavy metals were measured in urine, including total arsenic (As), arsenobetaine (Asb), dimethylarsinic acid (DMA), monomethylarsonic acid (MMA), barium (Ba), cadmium (Cd), cobalt (Co), cesium (Cs), mercury (Hg), lead (Pb), molybdenum (Mo), antimony (Sb), thallium (Tl), and tungsten (W). Arsenic is a metalloid rather than a heavy metal. DMA, Asb, and MMA are metabolites of As. However, As might induce toxic effects by combining and inactivating sulfhydryl enzymes similar to heavy metals (10, 25). Thus, we also listed As, Asb, DMA, and MMA as heavy metals according to previous studies (17, 26). Detailed information on the sample preparations and detection methods was summarized in Supplementary Table 1 and previously published elsewhere (27). The detection rate (%) and limit of detection (LOD) of heavy metals are presented in Supplementary Table 2. Metal concentration below the LOD was recorded as the LOD divided by the square root of two. Urinary creatinine was measured by Jaffé rate reaction before 2010 and Enzymatic Roche Cobas 6000 Analyzer in later research cycles.

### 2.3. Assessment of outcome

We used USFLI, a well-validated steatosis score, to define hepatic steatosis as a substitute for the liver biopsy (28, 29). The details of the calculation formula are presented in Supplementary material. Hepatic steatosis was defined to be present in the USFLI score ≥30 (29). This cut-off point has been previously validated with a sensitivity and specificity of 62 and 88%, respectively (30).

MAFLD was defined by the presence of hepatic steatosis, demonstrated by serologic score (USFLI ≥ 30), with at least one of the MAFLD components (4): overweight/obesity [body mass index (BMI) ≥25 kg/m^2^], diabetes mellitus [fasting glucose levels ≥7 mmol/L, or hemoglobin A1c (HbA1c) ≥6.5%, or 2-h post-load plasma glucose levels (2h-OGTT) ≥11 mmol/L], and metabolic dysfunction (at least two metabolic risk abnormalities). The diagnostic criteria of metabolic abnormalities are displayed in Supplementary material (31). NAFLD was defined as the USFLI ≥ 30 in the absence of viral hepatitis (HBV or HCV) and excessive consumption history of alcohol (alcohol consumption ≥30/20 g/d for men and women) (28). The difference between was summarized in Supplementary Table 3.

### 2.4. Statistical analysis

The proportions of categorical variables and median (inter-quantile range, IQR) of continuous variables are presented among comparison groups. We compared baseline characteristics using Chi-square tests for categorical variables and the Mann-Whitney *U*-test for continuous variables. Before association analysis, we adopted a covariate-adjusted standardization method to adjust for the urine dilution of urinary metal, which was generally applied by previous studies due to lower statistical bias than former methods (32, 33). In this approach, log-transformed creatinine was first regressed on the variables [race, gender, age (in years), and BMI were included in the present study] known to affect urine dilution (34). Then a ratio is produced by dividing observed creatinine values by the predicted creatinine values obtained from the previous model. Finally, we standardized metal concentration by dividing the biomarker concentration by this ratio. Binary logistic regression, Bayesian Kernel Machine Regression (BKMR), and quantile-based g-computation (QGC) were then applied to evaluate the associations below.

#### 2.4.1. Binary logistic regression

Binary logistic regression models were applied to evaluate the associations of individual metals with NAFLD, MAFLD, and its components. In regression models, dilution-adjusted heavy metals were modeled as continuous (Ln-transformed) and categorical (i.e., quartiles). A linear trend test was performed by modeling the categorized metals as ordinal variables. Given the sex difference in the prevalence of NAFLD and MAFLD (35), these association analyses were further stratified by sex.

#### 2.4.2. Bayesian kernel machine regression

BKMR was implemented to estimate the joint and potential non-linear association of metal exposure with MAFLD, NAFLD, and MAFLD components. BKMR is a statistical approach combining Bayesian and statistical learning methods to investigate mixed exposure-response functions using a Gaussian Kernel function (36). This approach is a non-parameter statistical method without hypothesis testing but visualizes the exposure-response associations of each chemical and the joint influence of all chemicals. The probit BKMR model was applied to binary outcomes. The core function formula in this study is presented as follows:


$$\begin{array}{l} {\text{Φ}}^{-\;1}(\text{P}({\text{Y}}_{\text{i}}\;=\;1))\;=\;\text{h}(\text{A}{\text{s}}_{\text{i}}\;+\;\text{As}{\text{b}}_{\text{i}}\;+\;\text{DM}{\text{A}}_{\text{i}}\;+\;\text{B}{\text{a}}_{\text{i}}\;+\;\text{C}{\text{d}}_{\text{i}}\;+\;\text{C}{\text{o}}_{\text{i}}\\ +\;\text{C}{\text{s}}_{\text{i}}\;+\;\text{H}{\text{g}}_{\text{i}}+\;\text{M}{\text{o}}_{\text{i}}\;+\;\text{P}{\text{b}}_{\text{i}}\;+\;\text{S}{\text{b}}_{\text{i}}+\;\text{T}{\text{l}}_{\text{i}}\;+\;{\text{W}}_{\text{i}})\;+\;{\text{x}}_{\text{i}}\beta \; \end{array}$$

Where Φ^−1^ is a probit link function and (Yi = 1) represents the probability of the relative outcome. Other covariates and their coefficients are denoted by xi and β, respectively. The function *h*() represents the exposure-response function considering the non-linear and interactive relationship between exposure and a latent continuous outcome (>0 equal to MAFLD or NAFLD, < 0 equal to non-MAFLD or non-NAFLD). A possible interpretation of *h*(z) in the probit BKMR model could be the correlation between metal exposures and a latent outcome. We applied the option of variable selection and 20000 iterations by the Markov Chain Monte Carlo algorithm. A posterior inclusion probability (PIP) was calculated to evaluate the relative importance of metal exposure to health outcomes (37). The BKMR results contain univariate exposure-response and cumulative mixture exposure relationships.

#### 2.4.3. Quantile-based g-computation

To validate the association of exposure to multiple metals with the outcomes, we employed the QGC for mixture analyses. QGC is a parameter-based statistical method that combines weighted quantile sum regression (WQS) and g-computation. Compared to WQS, QGC has particular advantages in allowing for directional heterogeneity and non-linear or non-additive effects of components of the mixture (38). This novel strategy was used to estimate the change in MAFLD, NAFLD, and MAFLD components risk for a synchronous one-quartile increase for all 13 heavy metals. The plot depicts heavy metal and health outcomes prediction at the joint exposure levels *via* g-computation and bootstrap variance with bootstrap up to 200.

#### 2.4.4. Covariates

We selected covariates based on a priori as potential confounders (20, 39, 40). Age at interview (“≥20 and <40,” “≥40 and <60,” “≥60”), gender (male, female), education (High school or less, College, Graduate or higher), race/ethnicity (Hispanic, Non-Hispanic White, Non-Hispanic Black, Other), smoking status, and physical activity were collected using self-administered questionnaires. The ratio of family income to poverty (PIR) was calculated by dividing annual family income by the poverty threshold and dichotomized (PIR < 1 and PIR ≥ 1) for analysis. Smoking status was grouped into three categories: current smoker (smoking at least 100 cigarettes in lifetime, and smoking every day or some days at the time of interview), former smoker (smoking at least 100 cigarettes in lifetime, but not smoking at the time of interview), and never smoker (having not smoked 100 cigarettes during life). The participants engaged in vigorous or moderate recreational activities were identified as having regular physical activity. Diabetes, hypertension, BMI, HDL cholesterol, and high TG were not adjusted due to application in MAFLD diagnosis. The samples were weighted to reduce the selection bias among subgroups for age, gender, and race/ethnicity in the NHANES survey. However, these variables for calculating sample weights are already included in the models, especially for BKMR and QGC models. Therefore, as recommended, we used unweighted estimation for the main results and logistic models incorporating sampling weights in the sensitivity analysis (22, 41, 42).

#### 2.4.5. Sensitivity analyses

To test the robustness of our results, we conducted several sensitivity analyses. First, we used the natural log-transformed heavy metals in regression models to validate the role of creatinine. Second, we reanalyzed the regression model accounting for sample design, sampling weights, and strata. NHANES selected representative participants using a complex, multistage, and probability sampling design. Specifying the sampling design parameters (including sample weights) should be considered to reduce biased estimates (43). For combing multiple survey cycles, the sample weight was calculated by “WTMEC2YR (variable name of weight)/*n* (the number of survey circles)” (43). SAS PROC SURVEYLOGISTIC was used for logistic regression analyses while incorporating survey design. BKMR and QGC methods do not support the survey design, and these analyses were limited to conventional binary logistic regression.

Statistics analyses were performed using SAS 9.4 (SAS Institute Inc., Cary, NC) and R 4.1.1 (44). BKMR and QGC were conducted using “bkmr” and “qgcomp” packages, respectively. The *P*-value was 0.05 for the significance level.

## 3. Results

### 3.1. Population characteristics

A total of 1,811 (32.6%) and 1,624 (34.2%) individuals were diagnosed with MAFLD and NAFLD, respectively. Participants with MAFLD were older, less educated, Hispanic, and more likely to be current/past smokers and less physical activity than those without MAFLD (Table 1). The characteristics of participants with NAFLD were similar to those with MAFLD. Cases with MAFLD or NAFLD had a higher prevalence of MAFLD components (i.e., diabetes, overweight/obesity, and metabolic dysfunction).

**Table 1:** Demographic characteristics of participants grouped by MAFLD and NAFLD, NHANES 2003–2018 (*N* = 5,548).

**Variables**	**Non-MAFLD**	**MAFLD**	***P^a^*** **-value**	**Non-NAFLD**	**NAFLD**	***P^a^*** **-value**
**Demographic variable**						
Age, *n* (%)			0.001			<0.001
≥20 and <40	1,470 (39.3%)	442 (24.4%)		1,225 (39.2%)	388 (23.9%)	
≥40 and <60	1,171 (31.3%)	617 (34.1%)		929 (29.7%)	523 (32.2%)	
≥60	1,096 (29.3%)	752 (41.5%)		972 (31.1%)	713 (43.9%)	
Sex, male, *n* (%)	1,727 (46.2%)	1,030 (56.9%)	< 0.001	1,379 (44.1%)	900 (55.4%)	<0.001
Ratio of family income to poverty, ≥1, *n* (%)	3,019 (80.8%)	1,429 (78.9%)	0.107	2,511 (80.3%)	1,272 (78.3%)	0.113
Education, *n* (%)			<0.001			<0.001
High school or less	1,663 (44.5%)	1,007 (55.6%)		1,414 (45.2%)	904 (55.7%)	
College	1,092 (29.2%)	527 (29.1%)		904 (28.9%)	478 (29.4%)	
Graduate or higher	982 (26.3%)	277 (15.3%)		808 (25.8%)	242 (14.9%)	
Race, *n* (%)			<0.001			<0.001
Hispanic	760 (20.3%)	643 (35.5%)		659 (21.1%)	591 (36.4%)	
Non-Hispanic White	1,664 (44.5%)	806 (44.5%)		1,348 (43.1%)	714 (44.0%)	
Non-Hispanic Black	885 (23.7%)	231 (12.8%)		750 (24.0%)	194 (11.9%)	
Other race	428 (11.5%)	131 (7.23%)		369 (11.8%)	125 (7.70%)	
Smoking, *n* (%)			<0.001			<0.001
Never	2,132 (57.1%)	877 (48.4%)		1,886 (60.3%)	827 (50.9%)	
Former	819 (21.9%)	595 (32.9%)		669 (21.4%)	513 (31.6%)	
Current	786 (21.0%)	339 (18.7%)		571 (18.3%)	284 (17.5%)	
Have regular physical activity, yes, *n* (%)	2,098 (56.1%)	765 (42.2%)	<0.001	1,738 (55.6%)	679 (41.8%)	<0.001
**MAFLD components, yes**, ***n*** **(%)**						
Diabetes	342 (9.15%)	625 (34.5%)	<0.001	317 (10.1%)	561 (34.5%)	<0.001
Overweight/obesity	2,097 (56.3%)	1,778 (98.2%)	<0.001	1,809 (58.0%)	1,549 (95.6%)	<0.001
Metabolic dysfunction	2,331 (62.4%)	1,791 (98.9%)	<0.001	1,976 (63.2%)	1,607 (99.0%)	<0.001
High C-reaction protein	1,120 (40.3%)	920 (66.7%)	<0.001	950 (41.0%)	837 (67.7%)	<0.001
Central obesity	1,545 (41.3%)	1,584 (87.5%)	<0.001	1,355 (43.3%)	1,400 (86.2%)	<0.001
Insulin resistance	971 (26.0%)	1,731 (95.6%)	<0.001	821 (26.3%)	1,557 (95.9%)	<0.001
Low HDL cholesterol	698 (18.7%)	759 (41.9%)	<0.001	610 (19.5%)	716 (44.1%)	<0.001
Hypertension	1,400 (38.3%)	1,128 (63.1%)	<0.001	1,177 (38.5%)	1,000 (62.5%)	<0.001
Prediabetes	1,588 (44.0%)	1,126 (76.4%)	<0.001	1,337 (44.5%)	1,018 (77.1%)	<0.001
High triglyceride	647 (17.5%)	798 (44.5%)	<0.001	529 (17.1%)	723 (45.0%)	<0.001

MAFLD, Metabolic-associated fatty liver disease; NAFLD, Non-alcoholic fatty liver disease; LDL, Low-Density Lipoprotein; HDL, High-Density Lipoprotein.

Significance

a P-value < 0.05 from Chi-square or Mann-Whitney U-test.

Among 14 metals in the present study, we excluded MMA from the association analyses because the detection rate was <50% (Supplementary Table 2). The distribution of urinary metals stratified by MAFLD and NAFLD was presented in Supplementary Table 4. The participants with MAFLD or NAFLD had significantly higher levels of heavy metals. The Spearman correlations among these heavy metals varied from weak (0.05) to strong (0.82), as presented in Supplementary Figure 2. The strongest correlations were detected between DMA and As (*r* = 0.82).

### 3.2. Associations of single metal exposure with MAFLD, NAFLD, and MAFLD components

The results from the binary logistic regression models adjusted for the covariates are shown in Table 2. We found a significant positive association between As [OR 1.09 (95%CI 1.03–1.16)], DMA [OR 1.17 (95%CI 1.07–1.27)], Ba [OR 1.22 (95%CI 1.14–1.30)], Co [OR 1.22 (95%CI 1.11–1.34)], Cs [OR 1.35 (95%CI 1.18–1.54)], Mo [OR 1.45 (95%CI 1.30–1.62)], Sb [OR 1.18 (95%CI 1.08–1.29)], Tl [OR 1.49 (95%CI 1.33–1.67)], and W [OR 1.23 (95%CI 1.15–1.32)] with MAFLD. For NAFLD, patterns of associations with heavy metals were similar to those of MAFLD, except for As, where no significant association between As and NAFLD was observed (Table 2). The significant linear trend of the associations with these two outcomes was also observed when heavy metals were modeled as quartiles (*P* trend < 0.05, Supplementary Figures 3, 4). Additionally, Cd was significantly associated with MAFLD compared in Q2 [OR 1.30 (95%CI 1.08–1.56)], Q3 [OR 1.43 (95%CI 1.18–1.74)], and Q4 [OR 1.25 (95%CI 1.01–1.54)] to Q1, respectively, suggesting a non-linear association. Sex-stratified analyses revealed that the associations of these heavy metals with MAFLD and NAFLD were generally similar (*P-*int > 0.05, Supplementary Table 5) except for Hg, with stronger associations observed among females (*P-*int < 0.001). Positive associations generally remained significant when no adjustment for urinary dilution was made (Supplementary Table 6, model 1) or the NHANES complex survey design, the sample weight, was incorporated (Supplementary Table 6, model 2).

**Table 2:** Association of the urinary heavy metals with MAFLD and NAFLD in all participants from NHANES 2003 to 2018.

**Metal**	**MAFLD, OR (95%CI)**	**NAFLD, OR (95%CI)**
Asb	1.03 (0.99, 1.07)	1.02 (0.98, 1.06)
As	**1.09 (1.03, 1.16)**	1.07 (1.00, 1.14)
Ba	**1.22 (1.14, 1.30)**	**1.22 (1.14, 1.31)**
Cd	1.08 (0.98, 1.19)	1.09 (0.98, 1.21)
Co	**1.22 (1.11, 1.34)**	**1.22 (1.10, 1.35)**
Cs	**1.35 (1.18, 1.54)**	**1.32 (1.14, 1.52)**
DMA	**1.17 (1.07, 1.27)**	**1.12 (1.02, 1.23)**
Hg	0.95 (0.89, 1.01)	**0.92 (0.86, 0.99)**
Mo	**1.45 (1.30, 1.62)**	**1.51 (1.34, 1.69)**
Pb	1.00 (0.91, 1.10)	0.98 (0.89, 1.09)
Sb	**1.18 (1.08, 1.29)**	**1.19 (1.08, 1.31)**
Tl	**1.49 (1.33, 1.67)**	**1.47 (1.30, 1.67)**
W	**1.23 (1.15, 1.32)**	**1.23 (1.14, 1.33)**

Estimates were presented as odds ratio (OR) and 95% confidence intervals (CIs). The models were adjusted for age, gender, race, research cycle, education level, smoking status, PIR, and physical activity. Bold font for P < 0.05.

The associations of heavy metals with MAFLD components are presented in Supplementary Figure 5. The positive associations of Co, Cs, Mo, Tl, and W with MAFLD were also observed for diabetes mellitus, overweight/obesity, and metabolic dysfunction, specifically central obesity, insulin resistance, and prediabetes. The positive associations of MAFLD with As, DMA, and Sb were also observed for diabetes mellitus, whereas positive associations with Ba were observed for overweight/obesity.

### 3.3. Associations of metal mixture exposure and MAFLD, NAFLD, and MALFD components

**Figure 1:**
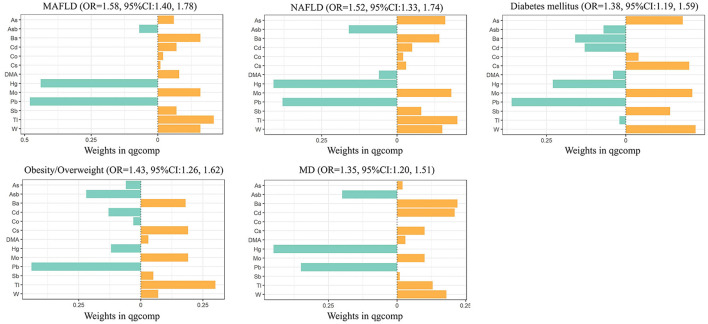
Combined association (95%CI) and qgcomp weights of metal mixture with MAFLD, NAFLD, and components of MAFLD by QGC models. Models were adjusted for age, gender, race, research cycle, education level, smoking status, poverty income ratio, and physical activity. Qgcomp, quantile g-computation; MAFLD, metabolic associated fatty liver disease; NAFLD, non-alcoholic fatty liver disease; MD, metabolic dysfunction.

The results of the QGC models showed that a quartile increase in the metal mixture was significantly associated with increased odds of being MAFLD [OR 1.58 (95%CI 1.40–1.78)]. As shown in Figure 1, Tl (0.21) contributed most to the positive association between heavy metals and MAFLD, followed by Mo (0.16), W (0.16), and Ba (0.16). Pb had the largest negative contribution to the overall effect, followed by Hg. We also observed a quartile increase in the QGC index was significantly associated with NAFLD [OR 1.52 (95%CI 1.33–1.74)], diabetes [OR 1.38 (95%CI 1.19–1.59)], overweight/obesity [OR 1.43 (95%CI 1.26–1.62)], and metabolic dysfunction [OR 1.35 (95%CI 1.20–1.51)]. Urinary Tl exposure was assigned the largest positive weights with NAFLD and obesity/overweight. Urinary W and Ba exposure were assigned to the strongest positive weights in the relationship with diabetes and metabolic dysfunction, respectively (Figure 1; Supplementary Figure 6).

**Figure 2:**
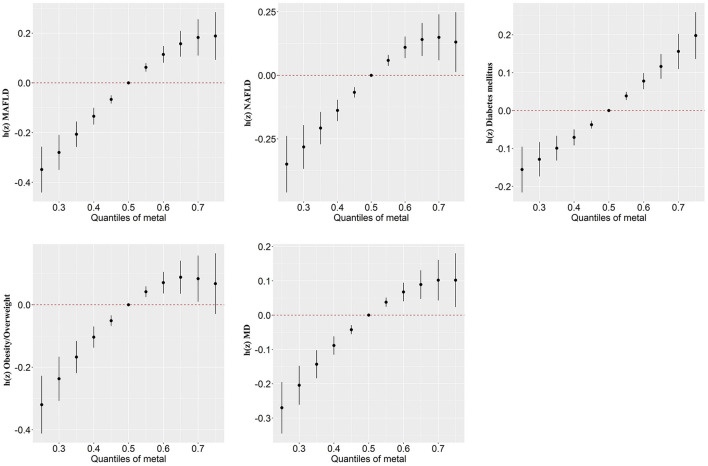
Combined association (95%CI) of metal mixture with MAFLD, NAFLD, and components of MAFLD by BKMR models, comparing all chemicals set at different levels with their 50th percentiles. Models were adjusted for age, gender, race, research cycle, education level, smoking status, poverty income ratio, and physical activity. BKMR, Bayesian kernel machine regression; MAFLD, metabolic associated fatty liver disease; NAFLD, non-alcoholic fatty liver disease; MD, metabolic dysfunction.

**Figure 3:**
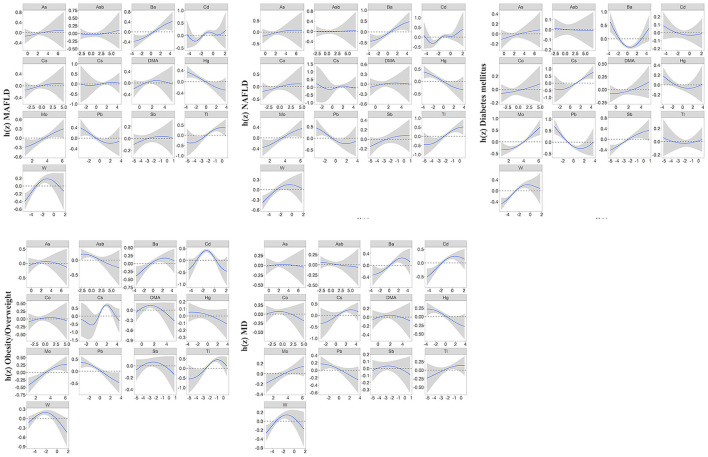
Univariate exposure-response function (95%CI) showed associations of heavy metals with MAFLD, NAFLD, and MAFLD components. All the remaining metal exposures are fixed at their median values. Results were adjusted for age, gender, race, research cycle, education level, smoking status, PIR, and physical activity. BKMR, Bayesian kernel machine regression; MAFLD, metabolic associated fatty liver disease; NAFLD, non-alcoholic fatty liver disease; MD, metabolic dysfunction; PIR, poverty income ratio.

In our study, the BKMR model was developed to estimate the combined effects of 13 urinary metal mixtures on MAFLD, NAFLD, and the component of MAFLD. Figure 2 presents the cumulative effect of the metal mixtures by comparing when all metals were at their 50th percentile and 95% confidence interval. The overall positive effects of metal mixtures on MAFLD and NAFLD were observed. Figure 3 displays the dose-response relationship with other metals set at median concentrations after adjusting for the covariates. Positive exposure-response relationships were observed between Ba, Mo, Tl, and W with MAFLD, while negative associations were observed for Hg and Pb. Similar patterns were observed for NAFLD. Additionally, the PIP for each metal was estimated (Supplementary Table 7). Among metal mixtures, the chemicals with the highest PIPs (1.00) were Ba, Cd, Hg, Mo, Pb, Tl, and W in the MAFLD model and Ba, Cd, Hg, Mo, Tl, and W in the NAFLD model.

Regarding the components of MAFLD, we observed a positive correlation of the overall metals mixture with diabetes, overweight/obesity, metabolic dysfunction, central obesity, prediabetes, insulin resistance, high C-reactive protein (CRP), and high triglyceride (TG) (Figure 2; Supplementary Figure 7). For single metal response, the exposure-response of Ba and Tl in MAFLD was similar to that of obesity/overweight and metabolic dysfunction (Figure 3; Supplementary Figure 8).

## 4. Discussion

Using multivariate logistic regression, BKMR, and QGC analysis of the metal mixture, we found that mixture of 13 analyzed metals was significantly associated with MAFLD, NAFLD, and the component of MAFLD. Tl, Mo, W, and Ba were positively associated with MAFLD based on logistic regression and BKMR. Tl, Mo, W, and Ba were also positively weighted in QGC. The positive weight of Tl and Ba on MAFLD may attribute to obesity/overweight and metabolic dysfunction, while that of Mo and W may mostly attribute to diabetes mellitus. Interestingly, urinary Hg and Pb were inversely associated with MAFLD or NAFLD risk in BKMR and QGC models.

Studies on the association between human exposure to individual and joint metals and MAFLD are sporadic. In both QGC and BKMR models, Tl, Mo, W, and Ba mainly contributed to the positive associations of heavy metal exposure with MAFLD risk. Findings in the single metal analysis of these metals also presented similarly positive associations. In line with our study, Asprouli et al. (45) reported a positive association of Tl with NAFLD risk or liver function indices among Greece's population. High Tl toxicity is mainly due to increased reactive oxygen species (ROS) and the interference of K-dependent reactions to secret insulin (46). Concerning Mo, decreased serum Mo was associated with a higher risk of NAFLD in Chinese males (47). The discordance in results may attribute to disparities in the study population or varied metrics used for the exposure assessment of metals. Excessive amounts of essential Mo may also cause toxicity by inducing the generation of reactive oxygen species (ROS) (48). For Ba and W, despite rare evidence from the population study, the hepatotoxicity of Ba and W was partially revealed by previous *in vivo* research. In the hepatocyte of rats, the high dose of Ba might increase the biomarkers with the implication of oxidative stress and disturb the activities of membrane-bound ATPases (49). The generation of ROS was similarly found in human liver cells exposed to an increased dose of W (48). Thus, more epidemiological studies are warranted to confirm the link between these metals and MAFLD.

In addition, to elucidate the MAFLD risk and heavy metal exposure, we also investigated associations with MAFLD components and heavy metals. The increased risk of MAFLD might be related to the development of diabetes mellitus, obesity/overweight, and metabolic dysfunction. Consistent results were shown by previous research. In U.S. adults or adolescents, combined impacts of metals, including Ba and Tl, were associated with obesity and type 2 diabetes (17, 50). Results from South Korea suggested the positive association between joint effects of Hg, Pb, and Cd with metabolic dysfunction, including hypertension, high TG, and central obesity (18). These findings indicated that exposure to heavy metals affects metabolic function, which is a significant risk factor for MAFLD and NAFLD.

Although the underlying mechanisms are not fully understood, both *in vivo* and *in vitro* studies provided valuable hints. Most metals, such as Cd, Ba, Tl, W, and As could induce ROS production, which in turn induces the release of apoptosis cytokine, activation of hepatic stellate cells, and finally, formation of fibrosis (51, 52). Meanwhile, the increased oxidative stress may also be generated by impaired homeostasis of essential trace elements, which act as important cofactors in many enzymes mediating such progress (53, 54). In rat liver mitochondria, Co could induce oxidative stress, in the presence of calcium, by highly damaging hydroxyl radical, finally resulting in apoptosis (55). Notably, such adverse effects are additive. For example, concurrent As and Cd exposure in rats is more damaging than separate exposure in triggering oxidant stress (56). Oxidant stress could also attribute to lipotoxic species, which are produced due to the overwhelmed disposal of fatty acids through beta-oxidation (57). In addition to ROS production, an essential factor advancing the development of fatty liver, heavy metals could directly disturb fatty acids' metabolism and increase fat accumulation in the liver (58). For example, Cd *in vivo* inhibits the fatty acid oxidation in the mitochondrial of hepatocytes, potentially through the sirtuin 1 signaling pathway (59). As could inhibit beta-oxidation of fatty acid by reacting with protein sulfhydryl groups and inactivating enzymes (10). The inactivation enzymes, less energy production, and more lipids production may also accelerate the development of MAFLD components. For example, Tl and Ba were associated with increased obesity, with similar mechanisms (17). By disrupting lipid metabolism, Cd could impair pancreatic β-cell function and exaggerate diabetes (60). Previous evidence showed that lipid accumulation could lead to hepatic insulin resistance and hepatic inflammation (61). Thus, the lipid metabolism disturbance could be a link between the comprehensive influence of metal exposure to fatty liver disease, obesity, diabetes, and other metabolic syndromes.

Our result indicated that urinary Pb and Hg were negatively associated with MAFLD risk, which was contrary to previous findings using blood biomarkers (62). We propose several potential explanations for this contradictory result. First, no significant association was observed between Hg and Pb with MAFLD in individual metal analysis. Thus, this inverse association might be attributed to complex antagonism between metals. The antagonistic interactions are common among metal mixtures due to the competition for carriers, metabolic interference, and morphological factors (63, 64). The adverse effects of these two metals on fatty liver disease might be alleviated in the metal mixture. Second, the various metrics (i.e., blood and urine) assessing the exposure level of Hg and Pb may be another possible reason. In U.S. populations, it is reported that the urinary Hg declined over the period 1999–2016, whereas there was a steady increase in blood organic Hg (65). Another research also suggested the difference between urinary and blood lead in analysis (66). However, the above hypothetical explanations need to be further validated by future studies.

Additionally, the inverse association between Hg with MAFLD and NAFLD was merely shown in men. Previous studies also reported the sex differences in health hazards of heavy metals. Heavy metals tend to correlate positively with NAFLD or liver fibrosis, more so in women than men (21, 22). Some hypotheses may explain this difference. For example, a lack of iron may contribute to the compensatory increase of heavy metal absorption by women (67). Rat models showed the expression of organic anion transporter (Oat) might also relate to the sex-specific organ toxicity of Hg exposure (68). In the male rat, the declined expression of Oat3 in the hepatocytes membranes after exposure would lower the intake of Hg, leading to a higher accumulation of Hg in the female liver. However, no sex disparity was found in other metals in our study, indicating future validation studies in a sex-specific manner.

We found significantly positive associations of the overall metal mixtures, including all 13 metals (As, Asb, DMA, Ba, Cd, Co, Cs, Hg, Pb, Mo, Sb, Tl, and W), with the risk of MAFLD or NAFLD, using BKMR and QGC models. Previous research on the combined associations of metal exposure on fatty liver disease is somewhat limited. Moon et al. utilized QGC to estimate the overall effects of Hg, Pb, and Cd on the hepatic steatosis index (HSI) and NAFLD risk (62). In this result among the Korean population, Elevated levels of Pb and Hg in total blood were associated with high HSI and increased risk of NAFLD. Similar results for the mixture of Hg, Pb, and Cd were reported by another study using the weighted quantile sum (WQS) regression model, QGC, and BKMR (69). In a cross-sectional study of Chinese males, the least absolute shrinkage and selection operator (LASSO) regression was used to explore the associations of 22 serum metals with NAFLD, which reported a negative association for Mo, and a positive association with Zn (47). Principle components analysis combined with Pearson correlation coefficients was also used to investigate multiple metal exposures, which suggested that the co-exposure to As-Hg, Pb-Cd, and Se-Zn pair patterns were linked to metabolic syndrome (70). Another two studies also used NHANES datasets for analysis. Li et al. used WQS and participants from NHANES 1999–2014 for analysis (23). Their results also showed a positive association between the metal mixture and NAFLD. Contrary to BKMR and QGC, WQS makes a unidirectionality assumption that all chemicals are positively or negatively associated with the given exposure (38) and this study merely incorporated the positive circumstance. Simultaneously, their study failed to take account of creatinine, an important marker affecting urinary concentrations of environmental contaminants (32). In another study using NHANES 2017–2020 (22), controlled attenuation parameter (CAP) and liver stiffness measurement (LSM) were utilized for the indicators of NAFLD and liver fibrosis. We used USFLI to diagnose NAFLD and MAFLD to include more participants because these two indices were merely available in two research cycles. No above studies applied the MAFLD or MAFLD components as the health outcomes.

BKMR and QGC models were used for multiple-metal exposure in this study. The BKMR model can resolve non-linear, multiple, and complex interactions between mixed exposures to metals or other chemicals (36). However, the BKMR model is not based on parametric inference. Similar to the BKMR model, QGC can address non-linear and non-additive effects but provide parametric inference results. Our research involved more diverse metals in the analysis and the final response function, compared with prior studies (47, 62, 69, 70). There are other established methods for analyzing mixture exposure in previous studies, including WQS, latent class analysis (LCA), and Lasso. Nevertheless, we were mainly interested in the overall association and interactions of all metals and chose BKMR and QGC for analysis.

There are several strengths of this study. First, to the best of our knowledge, this is the first study to illustrate the association between exposure to various heavy metals and MAFLD, the novel nomenclature form of fatty liver disease. Second, we explored the associations between heavy metals with MAFLD and NAFLD directly and with MAFLD components, serving as indirect evidence. Third, we used both BKMR and QGC models, which have complementary advantages. However, this study still has some notable limitations. First, this is a cross-sectional study, which does not allow the determination of temporality. Future prospective studies are required to investigate the causal relationships between joint metal exposure and MAFLD and NAFLD. Second, a definitive diagnosis of MAFLD and NAFLD by liver biopsy was unavailable from the NHANES database. Thus, we used USFLI to identify liver steatosis cases. However, compared with another generally used index, the hepatic steatosis index (HSI), USFLI was suggested to be more precise in the assessment of steatosis for the NHANES database (30).

## 5. Conclusion

In conclusion, this study suggested that exposure to metal mixtures is associated with the risk of MAFLD, NAFLD, and MAFLD components in US adults. Tl, Mo, W, and Ba contributed most to the MALFD risk, which could be instructive in MALFD prevention. Our findings apply the new definition of fatty liver disease and test its associations with risk factors in the real world. However, given the cross-sectional design of the present study, these findings are warranted to be confirmed by future longitudinal studies.

## Data availability statement

Publicly available datasets were analyzed in this study. This data can be found here: https://wwwn.cdc.gov/nchs/nhanes/Default.aspx.

## Ethics statement

The studies involving human participants were reviewed and approved by National Center for Health Statistics (NCHS) Institutional Review Board. The patients/participants provided their written informed consent to participate in this study.

## Author contributions

ZX and RA: methodology, data curation, formal analysis, visualization, writing—original draft, and writing—review and editing. MS: data curation. YQ: writing—review and editing and supervision. YJ: conceptualization, supervision, funding acquisition, and writing—review and editing. All authors approved the final version of the manuscript.
