# Supplementary material for: Associations of metal mixtures with metabolic-associated fatty liver disease and non-alcoholic fatty liver disease: NHANES 2003–2018
Source: Front Public Health. 2023 Mar 6;11:1133194. doi: 10.3389/fpubh.2023.1133194 (PMC10025549; doi:10.3389/fpubh.2023.1133194)
Supplement: Supplementary file 1 [file Data_Sheet_1.pdf]

frontiers

Supplementary Material

Associations of metal mixtures with metabolic-associated fatty liver disease and nonalcoholic fatty liver disease: NHANES 2003-2018

Zhilan Xie[1†], Ruxianguli Aimuzi[1†], Mingyu Si[1], Yimin Qu[1*], Yu Jiang[1*]

[1]School of Population Medicine and Public Health, Chinese Academy of Medical Sciences and Peking Union Medical College, Beijing, 100730, China

*** Correspondence:** Dr. Yu Jiang, School of Population Medicine and Public Health, Chinese Academy of Medical Sciences and Peking Union Medical College; E-mail address: jiangyu@pumc.edu.cn; or Dr. Yimin, Qu, School of Population Medicine and Public Health, Chinese Academy of Medical Sciences and Peking Union Medical College; E-mail address: quyimin@sph.pumc.edu.cn.

[†]These authors contributed equally to this work

1 Supplementary for assessment of outcome

We used USFLI, a well-validated steatosis score, to define hepatic steatosis as a substitute for the liver biopsy (1, 2). The score incorporates age, race/ethnicity, waist circumference, gamma-glutamyltransferase (GGT), fasting insulin, and fasting glucose. The formula of USFLI is as follows:

$$\mathrm{USFLI} = \frac{e^{-0.8073*\text{non-Hispanic black}+0.3458*\text{Mexican American}+0.0093*\text{age}+0.6151*\log_e(\text{GGT})+0.0249*\text{waist circumference}+1.1792*\log_e(\text{insulin})+0.8242*\log_e(\text{glucose})-14.7812}}{(1+e^{-0.8073*\text{non-Hispanic black}+0.3458*\text{Mexican American}+0.0093*\text{age}+0.6151*\log_e(\text{GGT})+0.0249*\text{waist circumference}+1.1792*\log_e(\text{insulin})+0.8242*\log_e(\text{glucose})-14.7812})} * 100$$

Hepatic steatosis was defined to be present in the USFLI score greater than or equal to 30 (2). This cut-off point has been previously validated with a sensitivity and specificity of 62% and 88%, respectively (3).

MAFLD was defined by presence of hepatic steatosis, demonstrated by serologic score (USFLI ≥30), with at least one of the MAFLD components (4): overweight/obesity (body mass index (BMI) ≥25 kg/m2), diabetes mellitus (fasting glucose levels ≥7 mmol/L, or hemoglobin A1c (HbA1c) ≥6.5%, or 2-h post-load plasma glucose levels (2h-OGTT) ≥ 11 mmol/L), and metabolic dysfunction (at least two metabolic risk abnormalities). Metabolic abnormalities included (5): (a) central obesity (waist circumference ≥102cm in men and ≥88cm in women) (b) hypertension (blood pressure ≥130/85 mmHg or prescription for hypertension); (c) High triglyceride (TG) (TG ≥150 mg/dL); (d) Low high-density lipoprotein (HDL) cholesterol (HDL ＜40/50 mg/dL in men and women); (e) Prediabetes (fasting glucose levels 5.6 to 6.9 mmol/L, or 2h-OGTT 7.8 to 11 mmol/L, or HbA1c 5.7% to 6.4%); (f) Insulin resistance (homeostasis model assessment of insulin resistance score (HOMA-IR)≥2.5); (g) high C-reactive protein (CRP) (CRP level ＞2 mg/L). NAFLD was defined as the USFLI ≥30, in the absence of viral hepatitis (HBV or HCV) and excessive consumption history of alcohol (alcohol consumption ≥30/20 g/d for men and women) (1).

Reference

1. European Association for the Study of the Liver (EASL); European Association for the Study of Diabetes (EASD); European Association for the Study of Obesity (EASO). EASL-EASD-EASO Clinical Practice Guidelines for the management of non-alcoholic fatty liver disease. J Hepatol. 2016 Jun;64(6):1388-402. doi: 10.1016/j.jhep.2015.11.004.
2. Ruhl CE, Everhart JE. Fatty liver indices in the multiethnic United States National Health and Nutrition Examination Survey. Aliment Pharmacol Ther. 2015 Jan;41(1):65-76. doi: 10.1111/apt.13012.
3. Barb D, Repetto EM, Stokes ME, Shankar SS, Cusi K. Type 2 diabetes mellitus increases the risk of hepatic fibrosis in individuals with obesity and nonalcoholic fatty liver disease. Obesity (Silver Spring). 2021 Nov;29(11):1950-1960. doi: 10.1002/oby.23263.
4. Eslam M, Newsome PN, Sarin SK, Anstee QM, Targher G, Romero-Gomez M, et al. A new definition for metabolic dysfunction-associated fatty liver disease: An international expert consensus statement. J Hepatol. 2020 Jul;73(1):202-209. doi: 10.1016/j.jhep.2020.03.039.
5. Chalasani N, Younossi Z, Lavine JE, Charlton M, Cusi K, Rinella M, et al. The diagnosis and management of nonalcoholic fatty liver disease: Practice guidance from the American Association for the Study of Liver Diseases. Hepatology. 2018 Jan;67(1):328-357. doi: 10.1002/hep.29367

Supplementary Figures and Tables

Supplementary table 1. Detection methods of heavy metal concentrations in urine, NHANES 2003-2018.

Metal	2003-2004	2005-2006	2007-2008	2009-2010	2011-2012	2013-2014	2015-2016	2017-2018
As	ICP-DRC-MS	ICP-DRC-MS	ICP-DRC-MS	ICP-DRC-MS	ICP-DRC-MS	ICP-DRC-MS	ICP-DRC-MS	ICP-DRC-MS
Asb	HPLC	HPLC	HPLC	HPLC	HPLC	HPLC	HPLC	HPLC
DMA	HPLC	HPLC	HPLC	HPLC	HPLC	HPLC	HPLC	HPLC
MMA	HPLC	HPLC	HPLC	HPLC	HPLC	HPLC	HPLC	HPLC
Ba	ICP-MS	ICP-MS	ICP-MS	ICP-MS	ICP-MS	ICP-MS	ICP-DRC-MS	ICP-DRC-MS
Cd	ICP-MS	ICP-MS	ICP-MS	ICP-MS	ICP-MS	ICP-MS	ICP-DRC-MS	ICP-DRC-MS
Co	ICP-MS	ICP-MS	ICP-MS	ICP-MS	ICP-MS	ICP-MS	ICP-DRC-MS	ICP-DRC-MS
Cs	ICP-MS	ICP-MS	ICP-MS	ICP-MS	ICP-MS	ICP-MS	ICP-DRC-MS	ICP-DRC-MS
Hg	Flow injection cold vapor atomic absorption analysis	ICP-DRC-MS	ICP-DRC-MS	ICP-DRC-MS	ICP-DRC-MS	ICP-DRC-MS	ICP-DRC-MS	ICP-DRC-MS
Mo	ICP-MS	ICP-MS	ICP-MS	ICP-MS	ICP-MS	ICP-MS	ICP-DRC-MS	ICP-DRC-MS
Pb	ICP-MS	ICP-MS	ICP-MS	ICP-MS	ICP-MS	ICP-MS	ICP-DRC-MS	ICP-DRC-MS
Sb	ICP-MS	ICP-MS	ICP-MS	ICP-MS	ICP-MS	ICP-MS	ICP-DRC-MS	ICP-DRC-MS
Tl	ICP-MS	ICP-MS	ICP-MS	ICP-MS	ICP-MS	ICP-MS	ICP-DRC-MS	ICP-DRC-MS
W	ICP-MS	ICP-MS	ICP-MS	ICP-MS	ICP-MS	ICP-MS	ICP-DRC-MS	ICP-DRC-MS

Abbreviations: ICP-DRC-MS, inductively coupled-plasma dynamic reaction cell-mass spectrometry; ICP-MS, inductively coupled plasma-mass spectrometry; HPLC, high-performance liquid chromatography.

Supplementary Table 2. Detection frequency of urinary metal by survey cycles among adults in NHANES 2003-2018

Metal	2003-2004		2005-2006		2007-2008		2009-2010		2011-2012		2013-2014		2015-2016		2017-2018		2003-2018
	LOD (ug/L)	n(%) >LOD	LOD (ug/L)	n(%) >LOD	LOD (ug/L)	n(%) >LOD	LOD (ug/L)	n(%) >LOD	LOD (ug/L)	n(%) >LOD	LOD (ug/L)	n(%) >LOD	LOD (ug/L)	n(%) >LOD	LOD (ug/L)	n(%) >LOD	n(%) >LOD
As	0.6	635 (98.4)	0.74	627 (98.4)	0.74	737 (98.7)	0.74	798 (99.2)	1.25	649 (97.0)	0.26	714 (99.1)	0.26	669 (99.2)	0.23	651 (99.6)	5480 (98.7)
Asb	0.4	473 (73.3)	0.4	500 (78.4)	0.4	449 (60.1)	0.4	541 (67.2)	1.19	342 (51.1)	1.16	365 (50.6)	1.16	348 (51.6)	1.16	316 (48.3)	3334 (60.0)
DMA	1.7	561 (86.9)	1.7	556 (87.2)	1.7	625 (83.7)	1.7	678 (84.3)	1.80	539 (80.5)	1.91	539 (74.8)	1.91	508 (75.3)	1.91	493 (75.4)	4499 (81.0)
MMA	0.9	223 (34.5)	0.9	234 (36.7)	0.9	262 (35.1)	0.9	261 (32.4)	0.89	203 (30.3)	0.20	526 (73.0)	0.20	410 (60.8)	0.20	310 (47.4)	2429 (43.7)
Ba	0.311	597 (92.5)	0.12	622 (97.6)	0.12	735 (98.5)	0.12	797 (99.1)	0.100	660 (98.6)	0.060	711 (98.7)	0.060	667 (98.9)	0.060	648 (99.2)	5437 (97.9)
Cd	0.042	625 (96.8)	0.042	610 (95.7)	0.042	725 (97.1)	0.042	784 (97.5)	0.056	620 (92.6)	0.036	696 (95.2)	0.036	651 (96.5)	0.036	641 (98.1)	5342 (96.2)
Co	0.085	609 (94.4)	0.041	630 (98.9)	0.041	740 (99.1)	0.041	796 (99.0)	0.048	663 (99.1)	0.023	714 (99.1)	0.023	668 (99.1)	0.023	652 (99.8)	5472 (98.6)
Cs	NA	640 (99.2)	NA	632 (99.2)	NA	742 (99.4)	0.066	799 (99.3)	0.120	665 (99.4)	NA	714 (99.1)	0.086	668 (99.1)	0.086	652 (99.8)	5512 (99.3)
Hg	0.141	548 (84.9)	0.085	610 (95.7)	0.085	654 (87.6)	0.085	772 (96.0)	0.057	647 (96.7)	0.127	569 (79.0)	0.127	441 (65.4)	0.127	371 (56.8)	3186 (57.4)
Mo	1.499	638 (98.9)	NA	632 (99.2)	0.919	741 (99.3)	0.920	799 (99.3)	0.990	665 (99.4)	0.800	714 (99.1)	0.800	669 (99.2)	0.80	652 (99.8)	5510 (99.3)
Pb	0.099	545 (84.4)	0.099	616 (96.7)	0.099	729 (97.7)	0.100	787 (97.8)	0.080	641 (95.8)	0.030	711 (98.7)	0.030	666 (98.8)	0.03	651 (99.6)	5346 (96.3)
Sb	0.028	356 (55.1)	0.032	526 (82.5)	0.033	566 (75.8)	0.032	591 (73.5)	0.041	374 (55.9)	0.022	538 (74.7)	0.022	540 (80.1)	0.022	527 (80.7)	4018 (72.4)
Tl	0.016	635 (98.4)	0.016	632 (99.2)	0.016	738 (98.9)	0.009	797 (99.1)	0.020	664 (99.2)	0.018	708 (98.3)	0.018	669 (99.2)	0.018	650 (99.5)	5493 (99.0)
W	0.014	492 (76.2)	0.021	569 (89.3)	0.021	663 (88.8)	0.021	714 (88.8)	0.026	544 (81.3)	0.018	554 (76.9)	0.018	565 (83.3)	0.018	561 (85.9)	4662 (840)

Abbreviations: LOD, limit of detection; NA, not applicable.

Supplementary table 3. Comparison of metabolic-associated fatty liver disease and nonalcoholic fatty liver disease diagnostic criteria.

	MAFLD	NAFLD
Identification of hepatic steatosis	Yes	Yes
Comorbidities with at least one of the metabolic disorders (overweight/obesity, diabetes mellitus, and metabolic dysfunction)	Yes	No
Exclusion of participants with a competing etiology of secondary hepatic steatosis (Excessive alcohol consumption or positive hepatitis B and C viral infection)	No	Yes

Supplementary Table 4. Distribution of urinary metals in the study population stratified by MAFLD and NAFLD, NHANES 2003-2018.

Metal (ug/L)	All participants	Non-MAFLD	MAFLD	*P* value	Non-NAFLD	NAFLD	*P* value
	Median (Q1, Q3)	Median (Q1, Q3)	Median (Q1, Q3)		Median (Q1, Q3)	Median (Q1, Q3)	
As	7.87 (4.10, 17.2)	7.77 (4.01, 17.3)	8.07 (4.34, 17.1)	0.208	7.46 (3.82, 16.4)	7.66 (4.12, 16.2)	0.390
Asb	1.27 (0.82, 6.53)	1.30 (0.82, 6.58)	1.22 (0.82, 6.33)	0.747	1.20 (0.82, 6.48)	1.13 (0.82, 5.97)	0.576
DMA	3.69 (2.12, 6.41)	3.59 (2.05, 6.30)	3.90 (2.25, 6.67)	0.007	3.50 (2.00, 6.16)	3.70 (2.15, 6.38)	0.039
MMA	0.64 (0.60, 0.99)	0.64 (0.60, 1.03)	0.64 (0.53, 0.84)	<0.001	0.64 (0.60, 1.01)	0.64 (0.51, 0.76)	<0.001
Ba	1.14 (0.58, 2.11)	1.10 (0.56, 2.04)	1.24 (0.62, 2.32)	<0.001	1.09 (0.56, 2.03)	1.22 (0.61, 2.34)	<0.001
Cd	0.29 (0.15, 0.53)	0.28 (0.14, 0.52)	0.31 (0.17, 0.54)	<0.001	0.28 (0.14, 0.52)	0.31 (0.17, 0.54)	<0.001
Co	0.34 (0.22, 0.53)	0.34 (0.21, 0.53)	0.35 (0.23, 0.52)	0.016	0.34 (0.21, 0.54)	0.36 (0.24, 0.53)	0.015
Cs	4.58 (2.84, 6.82)	4.48 (2.79, 6.77)	4.80 (2.99, 6.91)	0.003	4.47 (2.76, 6.70)	4.76 (3.01, 6.90)	0.003
Hg	0.39 (0.17, 0.88)	0.40 (0.18, 0.91)	0.35 (0.16, 0.82)	<0.001	0.40 (0.18, 0.91)	0.34 (0.15, 0.78)	<0.001
Mo	39.5 (22.0, 64.5)	38.1 (21.2, 63.7)	41.6 (24.1, 66.3)	<0.001	37.4 (20.9, 63.2)	41.8 (24.4, 66.4)	<0.001
Pb	0.46 (0.25, 0.82)	0.46 (0.24, 0.81)	0.47 (0.27, 0.87)	0.003	0.45 (0.23, 0.79)	0.47 (0.27, 0.83)	0.002
Sb	0.05 (0.03, 0.09)	0.05 (0.03, 0.09)	0.05 (0.03, 0.09)	0.013	0.05 (0.03, 0.09)	0.05 (0.03, 0.09)	0.006
Tl	0.16 (0.10, 0.26)	0.16 (0.10, 0.25)	0.17 (0.10, 0.26)	0.022	0.16 (0.10, 0.25)	0.17 (0.10, 0.26)	0.017
W	0.06 (0.03, 0.12)	0.06 (0.03, 0.11)	0.07 (0.04, 0.12)	<0.001	0.06 (0.03, 0.11)	0.07 (0.04, 0.12)	<0.001

Significance *P*-value <0.05 from Mann-Whitney U test.

Supplementary Table 5. Association of the heavy metal concentrations with MAFLD and NAFLD risk by gender

Metal	Male<br>OR (95%CI)	Female<br>OR (95%CI)	*P*-*int*
MAFLD			
Asb	1.07 (1.02, 1.13) *	0.99 (0.94, 1.05)	0.195
As	1.15 (1.06, 1.25) *	1.03 (0.94, 1.12)	0.113
Ba	1.18 (1.07, 1.29) *	1.26 (1.14, 1.39) *	0.852
Cd	1.09 (0.95, 1.25)	1.07 (0.93, 1.23)	0.197
Co	1.29 (1.11, 1.49) *	1.18 (1.03, 1.35) *	0.428
Cs	1.33 (1.11, 1.61) *	1.36 (1.12, 1.66) *	0.582
DMA	1.26 (1.11, 1.42) *	1.08 (0.95, 1.23)	0.220
Hg	1.00 (0.92, 1.10)	0.89 (0.81, 0.98)*	<0.001
Mo	1.35 (1.17, 1.56) *	1.58 (1.34, 1.86) *	0.379
Pb	0.95 (0.84, 1.08)	1.07 (0.93, 1.23)	0.600
Sb	1.17 (1.04, 1.33) *	1.18 (1.03, 1.36) *	0.788
Tl	1.38 (1.17, 1.62) *	1.61 (1.36, 1.91) *	0.160
W	1.17 (1.05, 1.29) *	1.30 (1.17, 1.44) *	0.256
NAFLD			
Asb	1.06 (1.00, 1.12)	0.97 (0.91, 1.03)	0.128
As	1.14 (1.04, 1.25) *	1.01 (0.92, 1.11)	0.081
Ba	1.19 (1.08, 1.32) *	1.25 (1.13, 1.39) *	0.646
Cd	1.07 (0.92, 1.25)	1.09 (0.94, 1.26)	0.079
Co	1.37 (1.16, 1.61) *	1.13 (0.99, 1.30)	0.108
Cs	1.31 (1.07, 1.60) *	1.32 (1.07, 1.62) *	0.736
DMA	1.23 (1.07, 1.40) *	1.03 (0.89, 1.19)	0.190
Hg	0.97 (0.88, 1.07)	0.87 (0.80, 0.96)*	0.003
Mo	1.36 (1.16, 1.59) *	1.69 (1.42, 2.01) *	0.249
Pb	0.92 (0.80, 1.06)	1.06 (0.92, 1.23)	0.277
Sb	1.16 (1.02, 1.32) *	1.22 (1.05, 1.42) *	0.811
Tl	1.39 (1.16, 1.66) *	1.54 (1.29, 1.84) *	0.310
W	1.16 (1.03, 1.29) *	1.30 (1.17, 1.45) *	0.330

Estimates were presented as odds ratio (OR) and 95% confidence intervals (CIs). Adjusts for age, race, research cycle, education level, smoking status, PIR, and physical activity. * Refers to P <0.05; P-int: P for interaction between urinary metals (continuous) and gender. In subgroup analysis, gender was not adjusted. * for P<0.05.

Supplementary Table 6. Associations of urinary metals with MAFLD and NAFLD among adults

	Model 1	Model 2
MAFLD		
Asb	1.03 (0.99, 1.07)	1.05 (1.00, 1.10) *
As	1.06 (1.01, 1.12)*	1.12 (1.04, 1.20) *
Ba	1.17 (1.10, 1.24) *	1.24 (1.12, 1.37) *
Cd	1.03 (0.96, 1.10)	1.18 (1.04, 1.34) *
Co	1.11 (1.03, 1.20) *	1.34 (1.18, 1.52) *
Cs	1.12 (1.02, 1.22) *	1.37 (1.13, 1.67) *
DMA	1.10 (1.02, 1.19) *	1.21 (1.09, 1.35) *
Hg	0.95 (0.90, 1.01)	0.95 (0.86, 1.04)
Mo	1.19 (1.10, 1.28) *	1.53 (1.33, 1.77) *
Pb	0.98 (0.91, 1.06)	0.98 (0.87, 1.11)
Sb	1.12 (1.03, 1.21) *	1.20 (1.08, 1.34) *
Tl	1.24 (1.13, 1.36) *	1.44 (1.23, 1.68) *
W	1.16 (1.09, 1.24) *	1.28 (1.16, 1.41) *
NAFLD		
Asb	1.02 (0.98, 1.06)	1.03 (0.98, 1.08)
As	1.05 (1.00, 1.12)	1.08 (1.00, 1.17)
Ba	1.19 (1.11, 1.27) *	1.23 (1.11, 1.37) *
Cd	1.04 (0.97, 1.13)	1.19 (1.04, 1.37) *
Co	1.13 (1.04, 1.22) *	1.33 (1.16, 1.53) *
Cs	1.13 (1.03, 1.25) *	1.32 (1.10, 1.60) *
DMA	1.09 (1.00, 1.18) *	1.14 (1.02, 1.28) *
Hg	0.94 (0.88, 0.99) *	0.93 (0.85, 1.02)
Mo	1.23 (1.13, 1.33) *	1.58 (1.34, 1.85) *
Pb	0.99 (0.91, 1.07)	0.96 (0.85, 1.08)
Sb	1.15 (1.05, 1.25) *	1.20 (1.05, 1.36) *
Tl	1.26 (1.14, 1.39) *	1.44 (1.23, 1.69) *
W	1.17 (1.10, 1.25) *	1.28 (1.15, 1.41) *

Note: Binary logistic regression models were applied. Models were age, gender, race, research cycle, education level, smoking status, PIR, and physical activity. * $P < 0.05$.

Model 1: Urinary metals modeled without correcting for urinary dilution.

Model 2: Urinary metal concentrations were covariate-adjusted, creatinine-standardized, and ln transformed, binary logistic regressions accounting for NHANES complex survey design.

Supplementary Table 7. Posterior inclusion probabilities (PIPs) for inclusion each metal into MAFLD, NAFLD, and MAFLD components models, using Bayesian kernel machine regression (BKMR) model.

Metal	MAFLD	NAFLD	Diabetes mellitus	Obesity/ Overweight	Metabolic dysfunction	High CRP	Central obesity	Prediabetes	Insulin resistance	Low HDL	Hypertension	High TG
As	0.88	0.85	0.21	0.78	0.77	0.97	0.48	0.06	0.50	0.72	0.00	0.65
Asb	0.75	0.92	0.01	0.90	0.68	0.95	0.54	0.03	0.27	0.30	0.00	0.52
Ba	1.00	1.00	0.99	1.00	1.00	1.00	0.93	0.74	1.00	0.76	0.23	1.00
Cd	1.00	1.00	0.20	1.00	1.00	1.00	1.00	1.00	0.83	1.00	0.06	0.87
Co	0.74	0.84	0.05	0.83	0.81	1.00	0.67	0.15	0.54	0.71	0.09	0.89
Cs	0.95	0.99	0.99	1.00	0.96	1.00	1.00	0.16	0.69	0.64	0.07	0.91
DMA	0.94	0.88	0.27	0.93	0.81	0.86	0.94	0.06	0.82	0.76	0.05	0.86
Hg	1.00	1.00	0.50	0.32	0.99	0.99	0.89	0.08	1.00	0.93	0.14	0.84
Mo	1.00	1.00	1.00	1.00	0.89	0.72	1.00	0.13	1.00	0.91	0.03	0.93
Pb	1.00	0.99	1.00	0.99	0.89	1.00	1.00	0.18	1.00	0.99	0.06	0.93
Sb	0.67	0.84	1.00	0.76	0.70	0.99	0.47	0.08	0.61	0.54	0.32	0.86
Tl	1.00	1.00	0.28	1.00	0.95	1.00	0.90	0.41	1.00	0.65	0.23	0.90
W	1.00	1.00	1.00	0.86	0.99	1.00	0.98	0.13	0.98	0.99	0.52	0.97

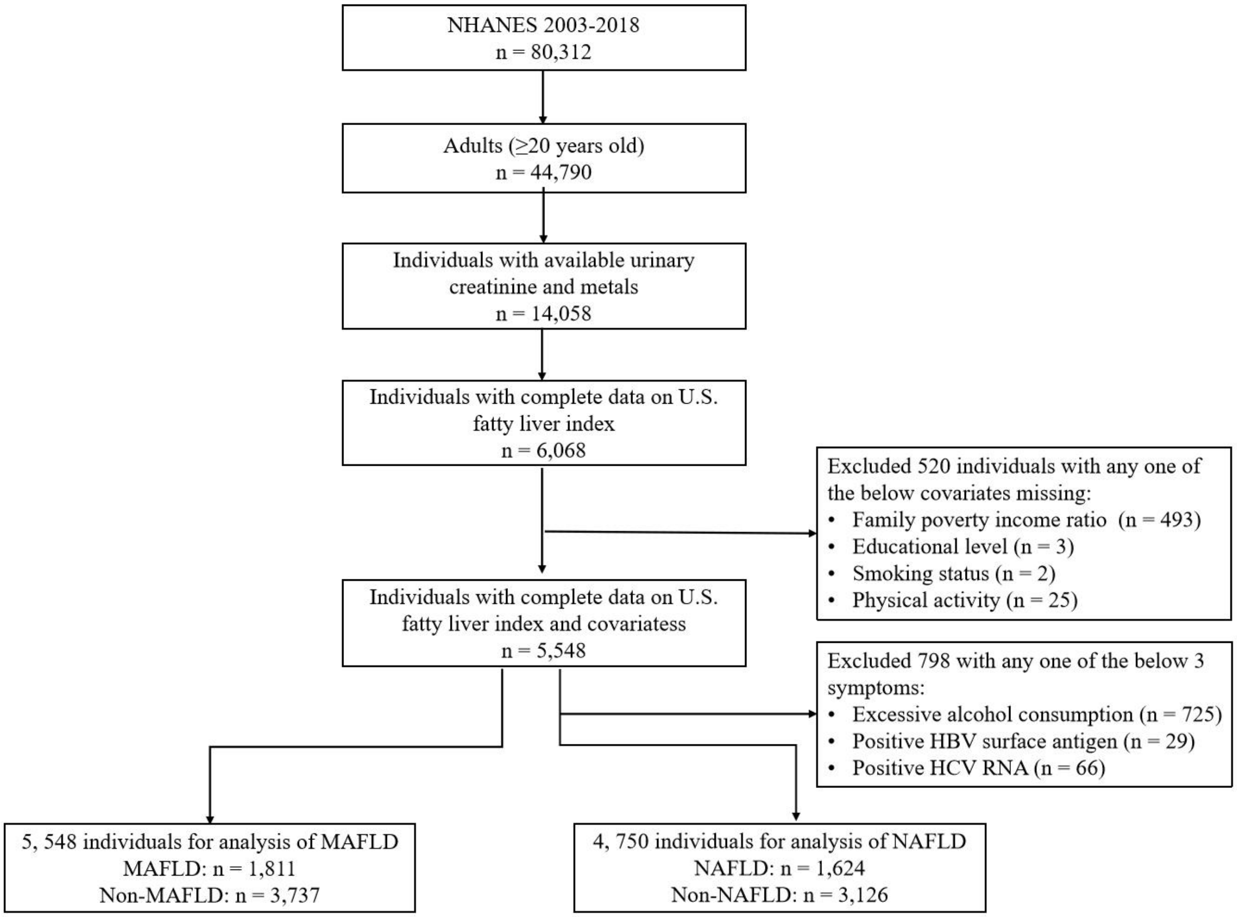

Supplementary Figure 1. The flow chart of inclusion and exclusion criteria based on the NHANES 2003-2018

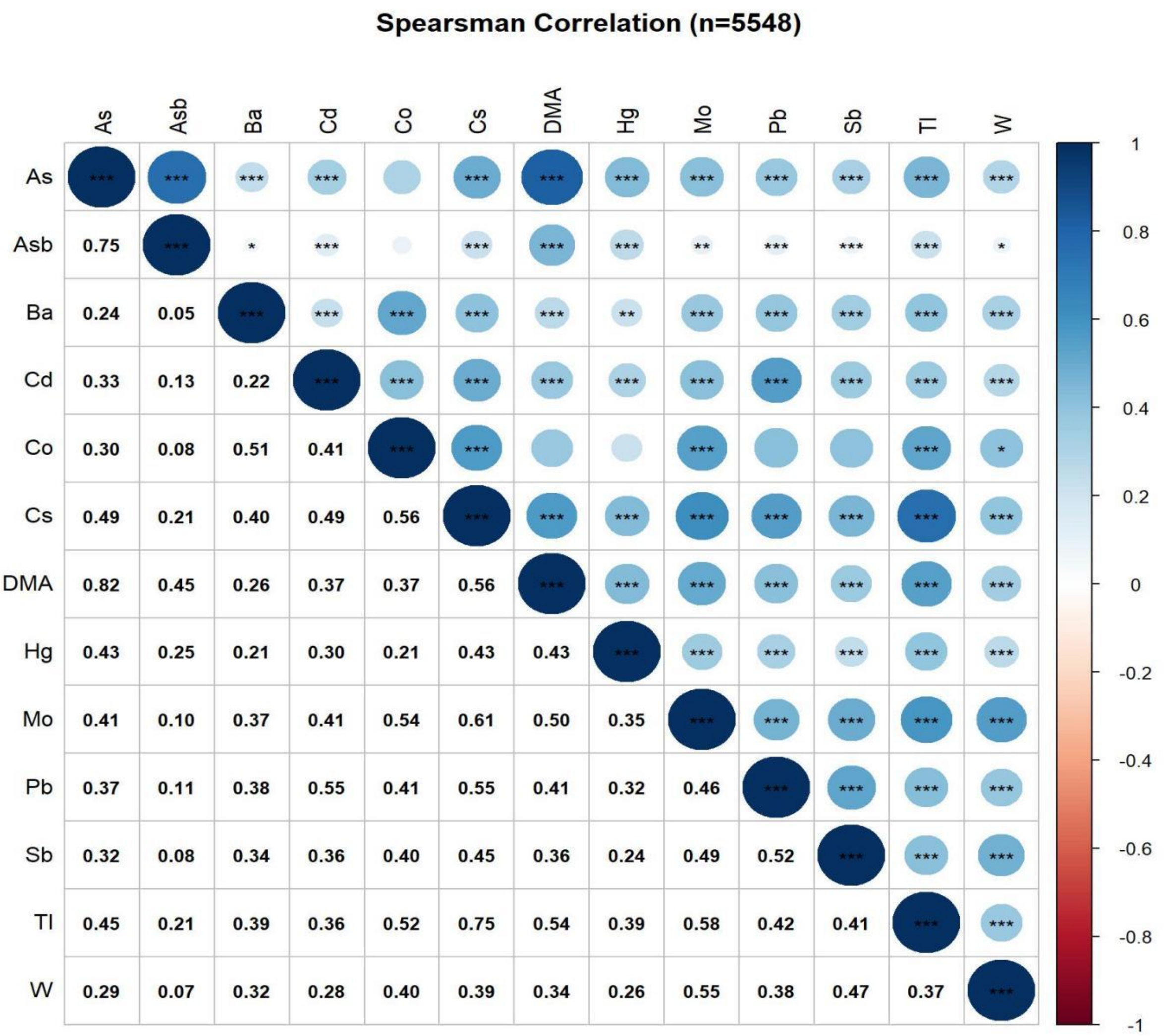

Supplementary Figure 2. Spearman correlations of urinary metal levels among adults in NHANES 2003-2018 (n = 5548). *$P < 0.05$; ** $P < 0.01$; *** $P < 0.001$.

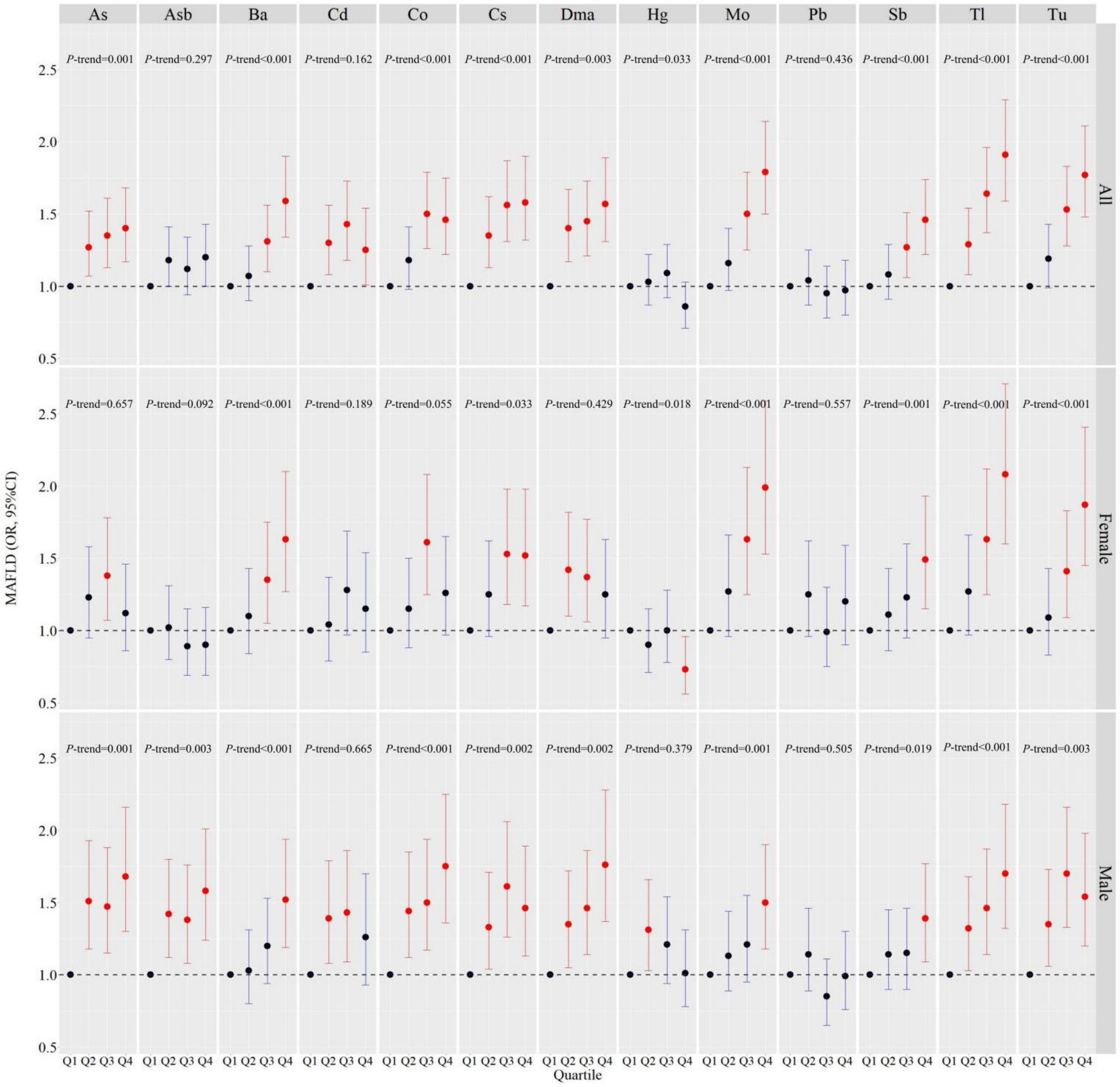

Supplementary Figure 3. Associations of urinary metal levels with MAFLD in adults. Binary logistic regression models were applied. Models were adjusted for age, gender, race, research cycle, education level, smoking status, PIR, and physical activity. Q1 to Q4 refers to the 1st to 4th quartiles of metals. The red point refers to P <0.05.

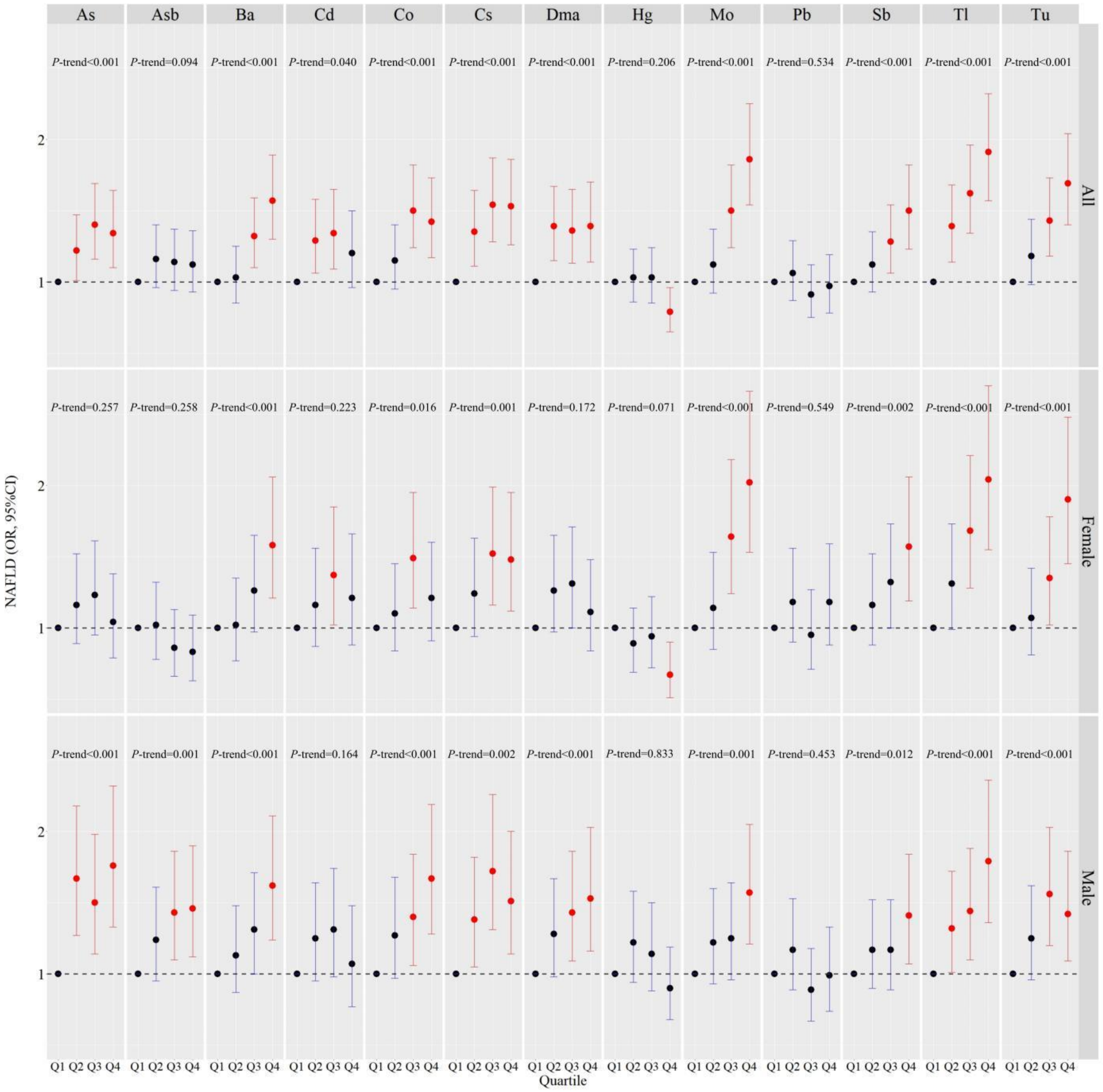

Supplementary Figure 4. Associations of urinary metal levels with NAFLD in adults. Binary logistic regression models were applied. Models were adjusted for age, gender, race, research cycle, education level, smoking status, PIR, and physical activity (only for all participants), smoking status, and physical activity. Q1 to Q4 refers to the 1st to 4th quartiles of metals. The red point refers to $P < 0.05$.

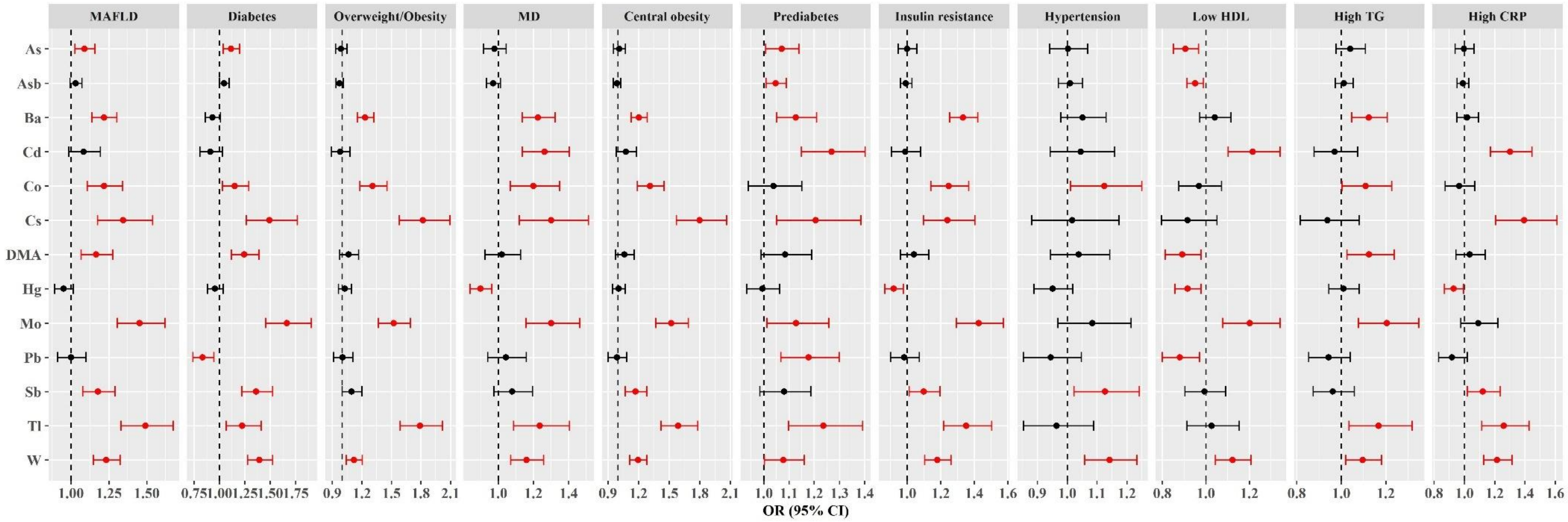

Supplementary Figure 5. Associations of urinary metals with MAFLD and its components in adults. Estimates were derived from binary Logistic regression models while adjusting for age, gender, race, research cycle, education level, smoking status, PIR, and physical activity. The red point refers to results with $P < 0.05$

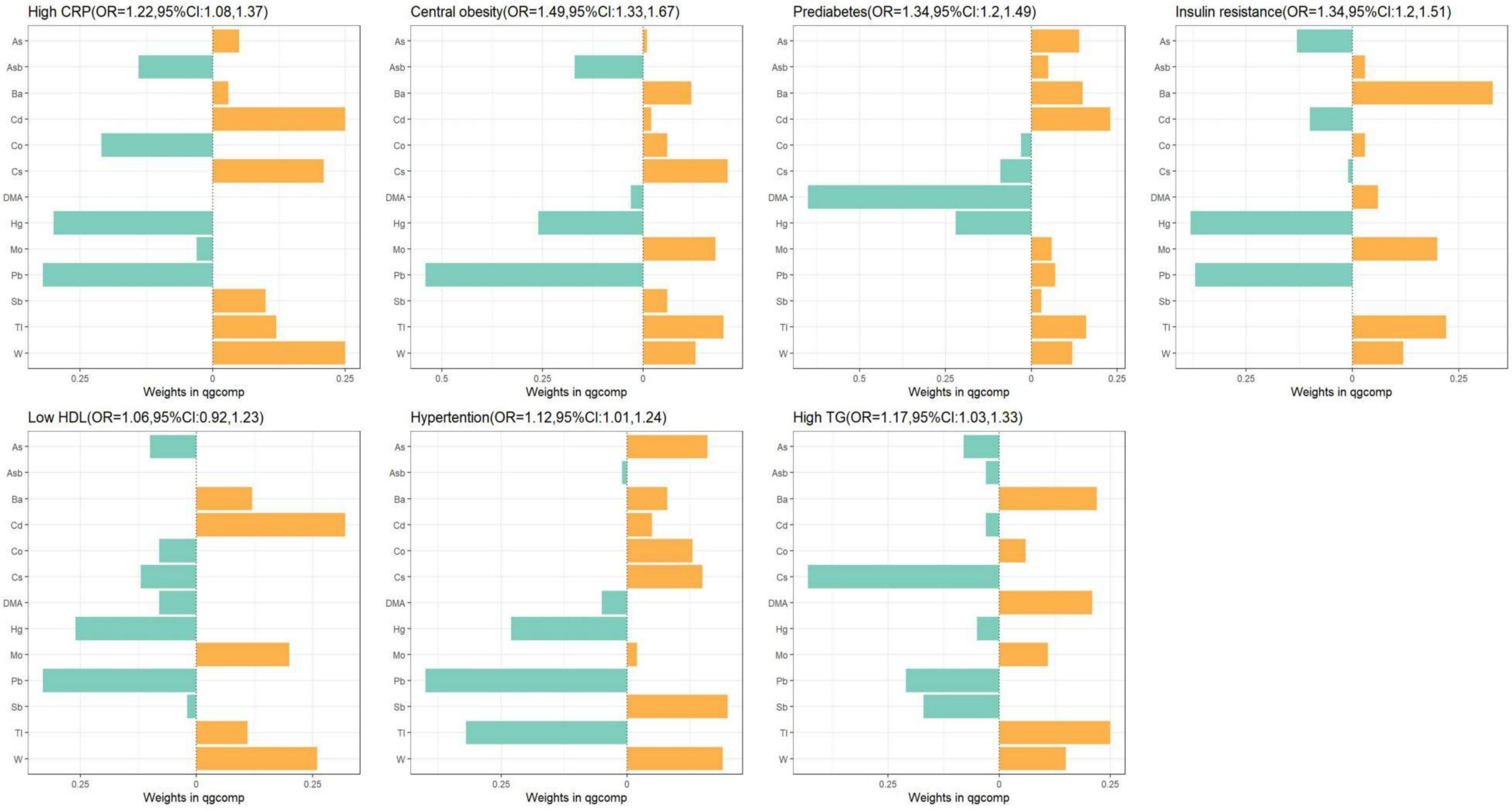

Supplementary Figure 6. Combined association (95%CI) and qgcomp weights of metal mixture with metabolic dysfunction by QGC. Models were adjusted for age, gender, race, research cycle, education level, smoking status, poverty income ratio, and physical activity. Qgcomp, quantile g-computation; CRP, C-reactive protein; HDL, high-density lipoprotein; TG, triglyceride.

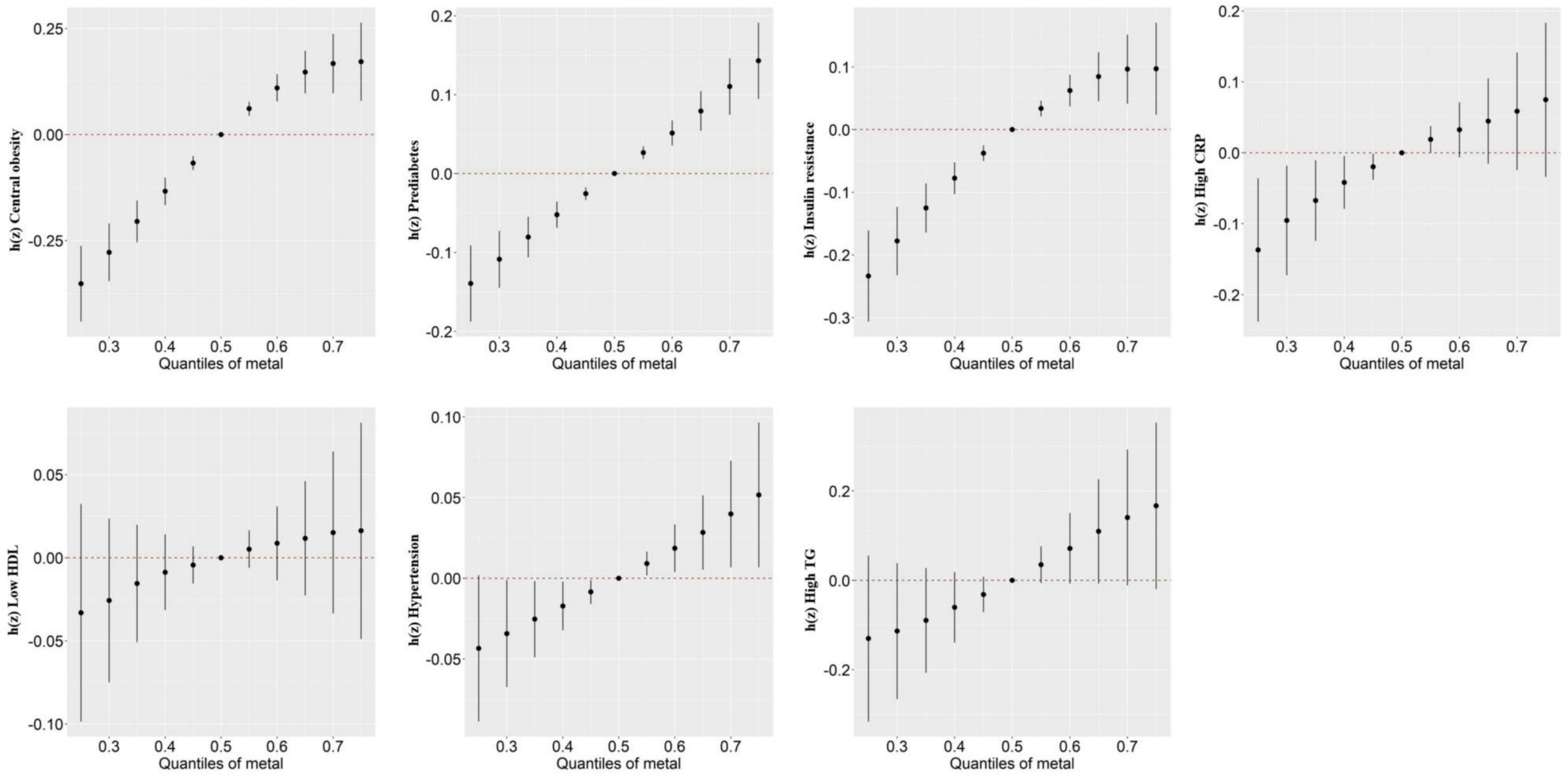

Supplementary Figure 7. Combined association (95%CI) of metal mixture with metabolic dysfunction by BKMR models, comparing all chemicals set at different levels with their 50th percentiles. Models were adjusted for age, gender, race, research cycle, education level, smoking status, poverty income ratio, and physical activity. BKMR, Bayesian kernel machine regression; CRP, C-reactive protein; HDL, high-density lipoprotein; TG, triglyceride.

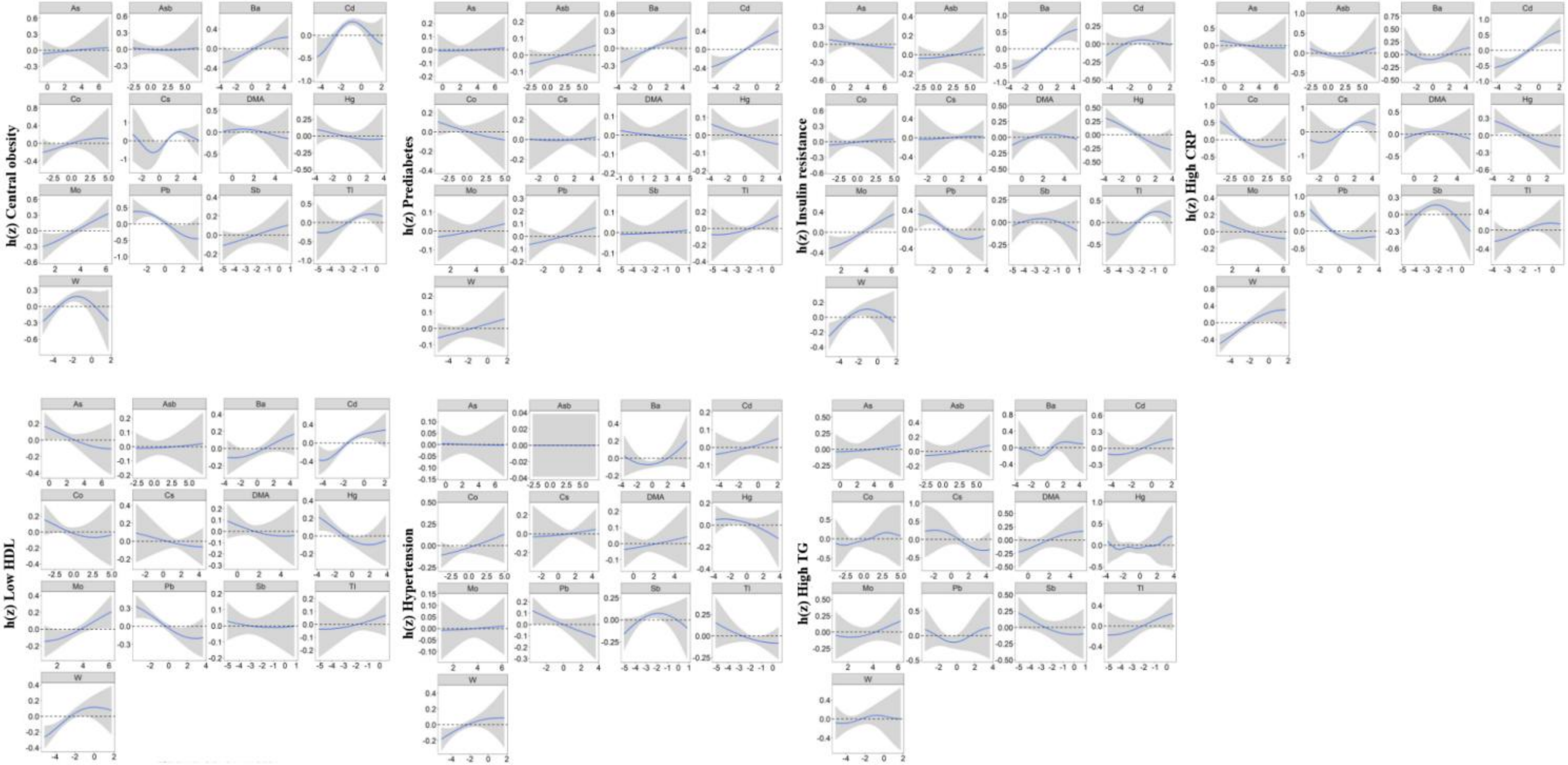

Supplementary Figure 8. Univariate exposure-response function (95%CI) showed associations between heavy metals and MAFLD components. All the remaining metal exposures are fixed at their median values. Results were adjusted for age, gender, race, research cycle, education level, smoking status, poverty income ratio, and physical activity. Note: BKMR, Bayesian kernel machine regression; CRP, C-reactive protein; HDL, high-density lipoprotein; TG, triglyceride.
